# Effects of different heat treatments on Maillard reaction products and volatile substances of camel milk

**DOI:** 10.3389/fnut.2023.1072261

**Published:** 2023-03-17

**Authors:** Xiaoxuan Zhao, Yinping Guo, Yumeng Zhang, Xiaoyang Pang, Yunna Wang, Jiaping Lv, Shuwen Zhang

**Affiliations:** Institute of Food Science and Technology, Chinese Academy of Agricultural Sciences, Beijing, China

**Keywords:** camel milk, flavor, furosine, 5-hydroxymethylfurfural, headspace-gas chromatography-ion migration spectrometry

## Abstract

Camel milk has unique compositional, functional and therapeutic properties compared to cow's milk and also contains many protective proteins with anti-cancer, anti-diabetic and anti-bacterial properties. In this experiment, fresh camel milk was heat-treated at different temperatures and times, and the changes in Millard reaction products were analyzed. Meanwhile, headspace-gas chromatography-ion migration spectrometry (HS-GC-IMS), electronic nose and electronic tongue were used to analyze the changes of volatile components in camel milk after different heat treatments. The results showed that the Maillard reaction was more severe with the increase of heat treatment, and the contents of furosine and 5-hydroxymethylfurfural increased significantly when the heat treatment temperature was higher than 120°C. HS-GC-IMS results showed that the contents of aldehydes and ketones increased obviously with the increase of heat treatment degree. The study clarifies the effects of different heat treatment degrees on Maillard reaction degree and flavor of camel milk, which has practical production guidance significance for the research and industrialization of liquid camel milk products.

## 1. Introduction

Interest in camel milk is spreading worldwide because of its unique composition, functional and therapeutic properties compared to cow milk (1). Camel milk has the essential amino acids required by human diet, and has a high proportion of β-casein, which is easy to hydrolyze, and lacks β-lactoglobulin, which is easy to induce allergy (2–4). In addition, camel milk also contains higher levels of vitamins and minerals than cow milk, and contains many protective proteins (such as immunoglobulin, lysozyme and lactoferrin) with anti-cancer, anti-diabetes and anti-bacterial properties (5–8).

Fresh milk contains harmful pathogenic bacteria such as *Escherichia coli, Salmonella* and *Staphylococcus aureus*. As the milk is rich in nutrients, it is easy to become a good breeding place for a variety of microorganisms. In order to ensure the safety of human consumption and prolong products shelf life, heat treatment is often used to sterilize dairy products (9). The more common heat treatments for dairy processing today include pasteurization, ultra-pasteurization and ultra-high temperature sterilization. However, excessive heat treatment can lead to Maillard reaction and denaturation of active protein, resulting in flavor and reduced nutrient content of camel milk. In the Maillard reaction process, the main reactants are lactose and lysine in casein and whey protein in camel milk. Different complex products will be generated at different stages, and with the increase of heat treatment degree, the content of harmful products will increase in different degrees (10). Maillard products such as furosine, furfurals, and advanced glycation end products can be potentially harmful to human health. Previous study reported to be associated with various types of inflammation and can lead to chronic diseases, including diabetes, kidney disease and Alzheimer's disease (11, 12). Furosine is a product of the initial stage of Maillard reaction and has been shown to be an indicator of heat damage during sterilization and storage. In addition, it is also one of the key indicators to identify reconstituted milk (13). 5-hydroxymethylfurfural is one of the intermediates of Maillard reaction and can be used to evaluate heat treatment damage of milk (14–16).

In addition, the flavor of camel milk after heat treatment will be different from that of raw camel milk, and the flavor changes will be different with the degree of thermal processing. Changes in microbial killing, cross-linking of whey proteins with casein, fat oxidation, and merad reactions resulting from heat treatment can all alter the flavor components in milk (17, 18). Nowadays, the research object of thermal processing of dairy products is mainly cow's milk, and the influence of thermal processing on camel's milk are limited.

In this study, furosine (FRS) and 5-hydroxymethylfural (5-HMF) content were measured to explore the degree of Maillard reaction in camel milk under different heat treatments. Meanwhile, combined with the electronic nose and electronic tongue technique to explore the flavor and taste differences of camel milk under different heat treatments, and the differences of volatile components in different camel milk samples were detected by headspace-gas chromatography-ion migration spectrometry (HS-GC-IMS). The heat treatment conditions are determined around 75°C for 15s, 120°C for 15s and 135°C for 5s, which are now the more common conditions for sterilizing dairy products.

## 2. Materials and methods

### 2.1. Sample collection and heat treatment

Raw camel milk sample was collected from Alashan, Inner Mongolia Farm, and is produced by different Bactrian camels, the final mixed collection of a total of sixteen L. Raw camel milk was divided into sixteen samples, the volume of each sample is one L. The control group was unheated samples, and the rest samples were treated with UHT pilot sterilizer (PT-20TS LAB TIT, POWERPOINT, Japan). By replacing heating tubes of different lengths, set the heat treatment time to 5, 15, and 30s, and each time corresponds to five heat treatment temperatures of 75, 90, 105, 120, and 135°C respectively. These three heating times and five heating temperatures were combined to form fifteen samples. Three replicates were done for each sample. Samples were stored at −80°C for further testing.

### 2.2. Detection of FRS by UPLC

The FRS content was measured according to the HPLC method described in the IDF standard 193/ISO 18329 (19) with some modifications. Two mL camel milk was absorbed into a heat-resistant sealed tube, and six mL of ten point six mol/L HCl was added. After mixing, the samples were heated at 110°C for twenty h for acid hydrolysis. After cooling, the samples were taken out and filtered with filter paper, and the filtrate was tested. Two mL sample hydrolysate was taken and protein content in sample solution was determined by Kjeldahl nitrogen determination method. One mL hydrolysate was mixed with five mL ammonium acetate solution of six g/L, and then filtered through a 0.22 μm aqueous phase membrane. Each sample was analyzed by UPLC (Waters-Acquit-Arc, Waters, America) using HSS T3 column (2.5 μm, 4.6 × 100 mm; Waters, MD, USA) at 35°C. Deionised water containing 0.1% trifluoroacetic acid was used as solvent A, and methanol was used as solvent B. In order to construct the standard curve, FRS standard solutions with mass concentrations of 25, 12.5, 6.25, 1.25, 0.625 and 0.125 μg/mL were prepared. The standard curve equation is y = 81,666x − 2,372.7 and the correlation coefficient R^2^ >0.99. The method has a detection limit of 0.3 μg/mL and a quantification limit of 0.9 μg/mL with a relative standard deviation of < 5.0%. All samples were analyzed in triplicate.

### 2.3. Detection of 5-HMF by UPLC

The method is based on Zhang et al. (20), and modified appropriately. Ten mL camel milk sample was added oxalic acid solution and mixed evenly. The mixed sample was then heated in boiling water for 25 mins. Then, ten mL methanol, three mL potassium ferrocyanide solution and three mL zinc acetate solution were added to the sample, mixed and stood for 30 min. Part of the supernatant was centrifuged, and the supernatant was filtered through a 0.45 μm filter membrane. UPLC (Waters-Acquit-Arc, Waters, America) with C18 columns (5 μm, 4.6 × 250 mm, Waters, America) was used for measurement. Methanol and deionised water were used as solvents A and B. In order to construct the standard curve, 5-HMF standard solutions with mass concentrations of 25, 12.5, 6.25, 1.25 and 0.625 μg/mL were prepared. The equation of the standard curve is y = 64,671.7x − 2,848.1 and the correlation coefficient R^2^ >0.99. The method has a detection limit of 0.005 μg/ml and a quantification limit of 0.015 μg/ml with a relative standard deviation of < 5.0%. All samples were analyzed in triplicate.

### 2.4. Detection of volatiles by electronic nose

Two mL of camel milk samples were placed in glass bottles for electronic nose analysis. The electronic nose adopts headspace injection method. The headspace acquisition time was sixty s, and the sampling delay was one hundred and eighty s. Information was collected at a relatively stable stage of forty-nine to fifty-two s. Principal component analysis (PCA) was performed on the electronic nose data. All samples were analyzed in triplicate.

### 2.5. Detection of volatiles by electronic tongue

After centrifugation at 4,500 r/min for 20 min, the fat was removed, then one mL skim milk was diluted with eighty mL ultrapure water. The diluted samples were placed in special beakers for testing. The samples were tested alternately with the calibration solution (ultrapure water), and each sample was tested for seven times. The data of the last four times which were relatively stable was selected for analysis. The electronic tongue data was analyzed by discriminant factor analysis (DFA).

### 2.6. Detection of volatile compounds by HS-GC-IMS

The volatile components were analyzed by HS–GC–IMS (FlavorSpec^®^, Gesellschaft für Analytische Sensorsysteme mbH, Dortmund, Germany). An analytical method slightly modified from Feng et al. (21), used as follows: two mL sample was placed in a twenty mL headspace sample vial, then the sample was incubated at 500 rpm and at 80°C for 20 min. Following this, a headspace volume of 500 μL was injected and injection needle temperature was 85°C. The GC equipped with a chromatographic column MXT-5 (0.1 μm, 15 m × 0.53 mm) was used for separation at 60°C, N_2_ (purity ≥99.999%) was used as carrier gas, and the initial carrier gas flow rate was set at two mL/min. It was maintained at two mL/min within zero to two min, and the carrier gas velocity increased linearly from two to ten mL/min within two to ten min. Then the carrier gas velocity linearly increases from ten to hundred mL/min in ten to twenty min. Finally, the carrier gas velocity linearly increases from hundred mL/min to one hundred and fifty mL/min in 20–30 min. The drift gas flow rate was set to one hundred and fifty mL/min. The column temperature was set at 60°C. GC-IMS analysis was conducted in triplicate. The retention index (RI) was calculated by a mixture of n-ketones (C4-C9). The GC-IMS Library Search used two-dimensinal cross-qualitative method for qualitative analysis, one of which was RI (National Institute of Standards and Technology database), and the other was Drift Time (IMS database).

### 2.7. Statistical analyses

Data are expressed as the means ± standard deviations (SD). The generate chart and radar charts were generated with Origin 2018, and statistical analysis was performed by SPSS Statistics 23 (*p* < 0.05 means the difference was significant). To better observe the significant changes of FRS and 5-HMF contents with the two independent variables of temperature and time, respectively, two independent one-way analyses were used for the analysis of FRS and 5-HMF data in the paper. Principal component analysis (PCA) and discriminant factor analysis (DFA) were used to distinguish different samples. Where PCA was analyzed using WinMuster 1.6.2.18 software. The DFA was analyzed using the software that comes with the Asrree II/LS16 electronic tongue instrument. Because the electronic tongue is more sensitive, the measured data instrument error is larger, the use of DFA can effectively reduce the instrument error, but PCA can not. In addition, HS-GC-IMS collected and analyzed only raw camel milk, heated at 75°C for 15 s, 120°C for 15 s, and 135°C for 5 s. This is because these three heating temperatures are commonly used for commercially available dairy products and it was desired to mainly observe the differences in volatile compounds between these three temperatures and raw camel milk.

## 3. Results and discussion

### 3.1. Changes in FRS and 5-HMF contents

In this experiment, FRS was measured in the raw camel milk: 6.89 mg/100 g protein, but no 5-HMF content was detected. From the results of the experiments in Table 1, FRS content increases slowly with the extension of heating time when the heating temperature was 75, 90, and 105°C, and the heating temperature had a great influence on FRS content. However, when the heating temperature was 120 and 135°C, both the heating temperature and time had a significant influence on FRS content. A similar change in FRS content can be seen in cow milk. When the heat treatment time was the same, the FRS content of cow milk samples was significantly different with the increase of heating temperature. As can be seen from Table 1, the FRS content of camel milk under the same heating conditions is lower than that of cow milk. This may be due to the lower lactose content in camel milk than in cow milk, or to the partial conversion of FRS to intermediate stage product 5-HMF. Compared with 75°C heating for 5 s, 15 s and 30 s, when the heat treatment temperature reached to 135°C, the FRS content of heat treated camel milk increased significantly, which was 7.1, 6.3, and 8.0 folds than of, respectively.

**Table 1:** Comparison of furosine and 5-hydroxymethylfural contents in camel milk and milk with different heat treatment degrees.

**Species**	**Compound**	**Time**	**Temperature**				
			**75**°**C**	**90**°**C**	**105**°**C**	**120**°**C**	**135**°**C**
Camel milk	FRS(mg 100 g^−1^ proteins)	5 s	7.39 ± 0.15^Aa^	9.08 ± 0.35^Ba^	16.26 ± 0.47^Cb^	21.50 ± 0.15^Da^	52.25 ± 0.40^Ea^
		15 s	9.14 ± 0.18^Ab^	10.81 ± 0.15^Bb^	15.09 ± 0.18^Ca^	33.44 ± 0.22^Db^	57.59 ± 0.09^Eb^
		30 s	10.48 ± 0.22^Ac^	11.04 ± 0.17^Bb^	15.57 ± 0.31^Cab^	46.61 ± 0.33^Dc^	83.86 ± 0.05^Ec^
	5-HMF(mg kg^−1^)	5 s	ND	ND	0.35 ± 0.001^Aa^	0.67 ± 0.02^Ba^	1.79 ± 0.03^Ca^
		15 s	ND	0.27 ± 0.02^Aa^	0.41 ± 0.01^Bb^	0.96 ± 0.02^Cb^	4.52 ± 0.01^Db^
		30 s	ND	0.3 ± 0.01^Aa^	0.38 ± 0.01^Bab^	1.39 ± 0.01^Cc^	4.56 ± 0.07^Db^
Cow milk (20)	FRS(mg 100 g^−1^ proteins)	5 s	17.4 ± 2.4^Aa^	23.8 ± 1.9^ABa^	32.4 ± 0.7^Ba^	61.7 ± 1.5^Ca^	153.6 ± 7.0^Da^
		15 s	24.6 ± 0.2^Ab^	27.4 ± 0.9^ABb^	38.9 ± 5.0^Bb^	67.7 ± 0.7^Cb^	166.8 ± 13.8^Db^
		30 s	25.3 ± 1.8^Ab^	28.9 ± 1.3^ABb^	39.2 ± 3.7^Bb^	111.8 ± 7.2^Cc^	255.3 ± 19.7^Dc^
	5-HMF(mg kg^−1^)	5 s	0.17 ± 0.003^Aa^	0.20 ± 0.022^ABa^	0.30 ± 0.033^Ba^	0.44 ± 0.030^Ca^	1.11 ± 0.043^Da^
		15 s	0.21 ± 0.005^Ab^	0.28 ± 0.048^ABb^	0.31 ± 0.058^Bb^	0.5 ± 0.089^Cb^	1.42 ± 0.072^Db^
		30 s	0.21 ± 0.044^Ab^	0.24 ± 0.013^ABc^	0.37 ± 0.025^Bc^	0.56 ± 0.023^Cc^	1.65 ± 0.119^Dc^

^a^Values followed by different uppercase and lowercase superscript letters are statistically different in heating time for each substance and in temperature for each substance, respectively (P < 0.05). Uppercase letters indicate the significance comparison of different temperatures at the same time; lowercase letters indicate the significance comparison of the same temperature at different times (P < 0.05). ND, not detected.

FRS is an early product of Maillard reaction and has been shown to be an indicator of heat damage during sterilization and storage (13). It can accumulate in milk after heat treatment (22). Moreover, the initial Maillard reaction products can be quantified indirectly by detecting FRS content (23). Since furosine is the product of the reaction between bovine milk protein and lactose after one of the products of the reaction after heat treatment. Therefore the formation of furosine is necessarily related to the temperature and time of the heat treatment. This is consistent with the results of this experiment. The results showed that the formation of FRS in camel milk was closely related to heating temperature and heating time. The increase of temperature will aggravate the reaction process, and the extension of time is an accumulation process of the reaction. It has been reported that the content of FRS in raw milk and low temperature pasteurized milk, extended shelf life pasteurized milk and UHT directly heated milk ranges from 3–5 mg/100 g, 4–29 mg/100 g, 8.2–78.3 mg/100 g and 48–300 mg/100 g, respectively (24–27). The results of the present study were consistent with those reported by previous.

When the heat treatment temperature was low and the heating time was short, the content of 5-HMF in camel milk was small or undetectable. However, 5-HMF can be detected in cow milk under lower heating conditions. As shown in Table 1, the content of 5-HMF was not detected in camel milk after heat treatment at 75°C for 5 s, 15 s, 30 s and 90°C for 5 s. When the heat treatment temperature was 105°C, the content of 5-HMF in camel milk increased slowly with the extension of heating time. When the heating temperature was increased to 120°C, compared with 105°C, the content of 5-HMF in camel milk increased significantly, and when the temperature was increased from 120°C to 135°C, the content of 5-HMF in camel milk increased sharply. This result also holds true for the data in Table 1 for milk. When the heating temperature was 135°C and the heating time was increased from 5 s to 15 s, the 5-HMF content of camel milk increased by more than 1.5 times, and the 5-HMF content in milk increased by 0.3 times.

The 5-HMF is a landmark product of Maillard reaction (14). The formation of 5-HMF in milk is related to the degree of thermal action. When the heat treatment temperature was reached to 135°C and heated for 15 s, the content of 5-HMF in camel milk increased significantly, which may be caused by the fact that ε-amino of protein lysine residue can also participate in the Maillard reaction of carbonyl group at higher temperature and longer heating condition (28). The higher 5-HMF content of camel milk than cow milk after heating under certain heating conditions may be due to the higher lysine content of camel milk than cow milk. Meng Gao (29) likewise found that the 5-HMF content in both pasteurized and UHT milk changed significantly with increasing heat treatment temperature and time, with higher temperatures and longer time periods resulting in higher 5-HMF content. Therefore, the Maillard reaction intensified, and the corresponding 5-HMF content also increased significantly.

### 3.2. Changes in electronic nose volatile characteristics

**Figure 1:**
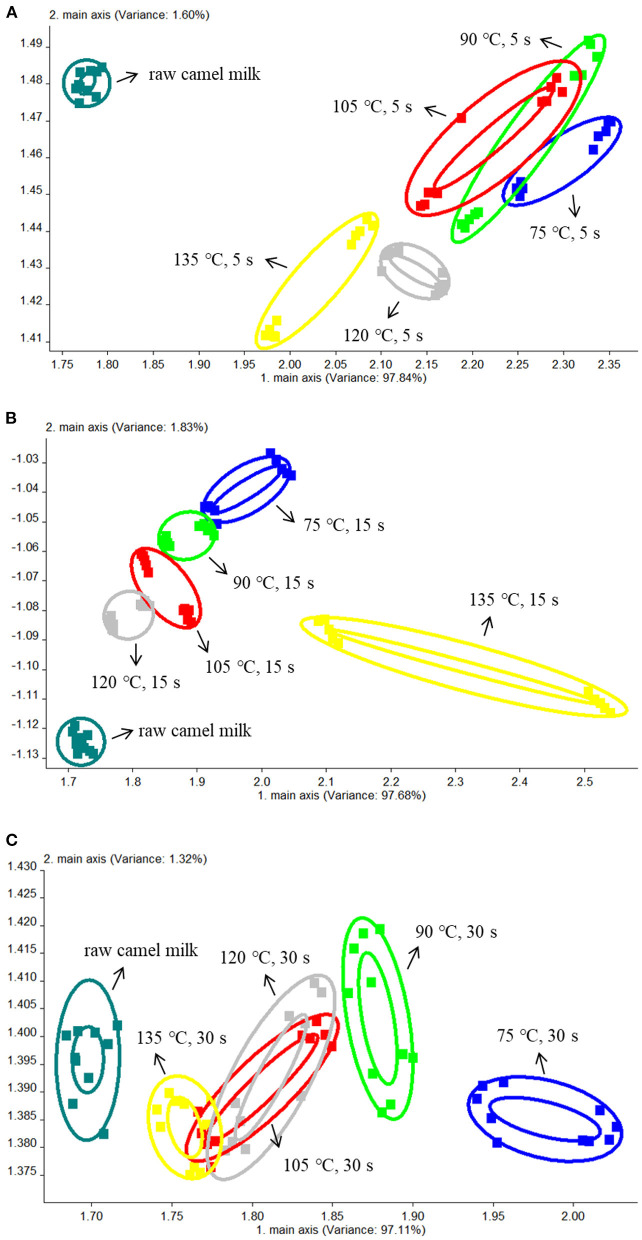
The PCA results of the electronic nose data from camel milk samples heated at different temperatures for 5 s **(A)**, 15 s **(B)**, and 30 s **(C)**.

The electronic nose used PCA to extract multi-index information, and then converts and reduces dimension data (30). Figures 1A–C were the comparative analyses of samples treated at different temperatures for 5, 15 and 30 s respectively. The total contribution rate of PC1 and PC2 was 99.44, 99.51, and 98.43% respectively, all of which were >95%. Therefore, PC1 and PC2 represent the main information features. The ellipse in the figure represents the repeated overall information characteristics of a single sample, and the distance of the figure represents the size of the odor difference between samples. As shown in Figure 1A, there was no significant difference between the samples heated for 5 s at 75, 90, and 105°C, the odor among the three groups of samples was relatively similar. However, when the heating temperature was increased to 120 and 135°C, it could be seen from the figure that there was no coincidence with the other four groups, indicating that there was significant difference in odor compared with the other four groups. In Figure 1B, the 6 groups of samples were relatively dispersed, and there were significant differences among the other samples except for the samples heated at 90°C and 105°C. In Figure 1C, only the samples heated at 105°C and 120°C showed no significant difference when heated for 30 s. It can be seen from A, B, and C that there were significant differences between raw camel milk and heat-treated camel milk.

### 3.3. Changes in electronic tongue volatile characteristics

**Figure 2:**
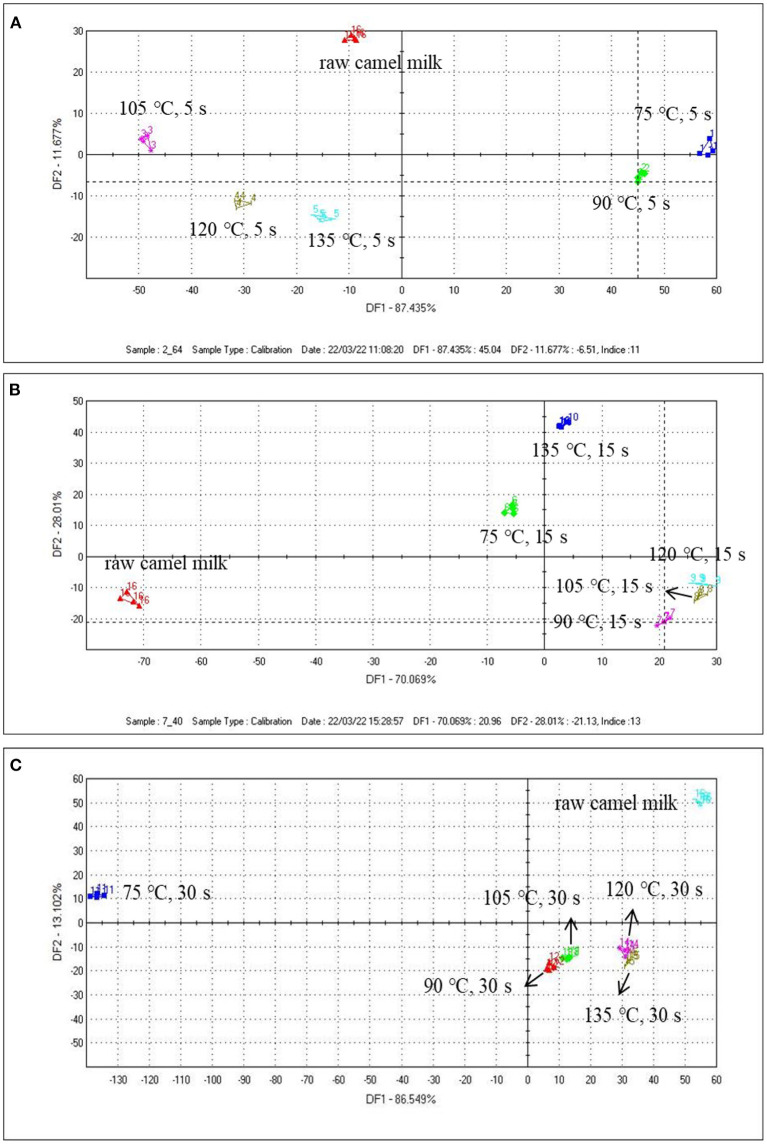
The DFA results of the electronic tongue data from camel milk samples heated at different temperatures for 5 s **(A)**, 15 s **(B)**, and 30 s **(C)**.

The ability of the electronic tongue to discriminate camel milk from different heated temperatures of the same heated time (5, 15, and 30 s) was studied. It can be seen from the figures that the repeatability of the sample was very good, which can reflect the overall characteristic information of the sample. Among them, the first discriminant factor DF1 was the main factor producing the difference of the four samples, which provides a great contribution to the taste of difference of the samples. In Figure 2A, the samples were independent and did not overlap with each other. There were significant differences among the samples. As can be seen from the figure, samples with heat treatment temperature of 75 and 105°C can be separated by DF1. In Figure 2B, it can be seen from DF1 that the distance between 105 and 120°C was the smallest, which indicated the taste difference between the two samples was relatively close, but once the heating temperature rises to 135°C, the taste of camel milk will be greatly affected. When the heating time was extended to 30 s (Figure 2C), except for the raw camel milk and the sample heated at 75°C, the remaining four groups of samples were relatively similar, and the samples heated at 120 and 135°C were partially overlapped, indicating that there was little difference in taste between the two groups.

**Figure 3:**
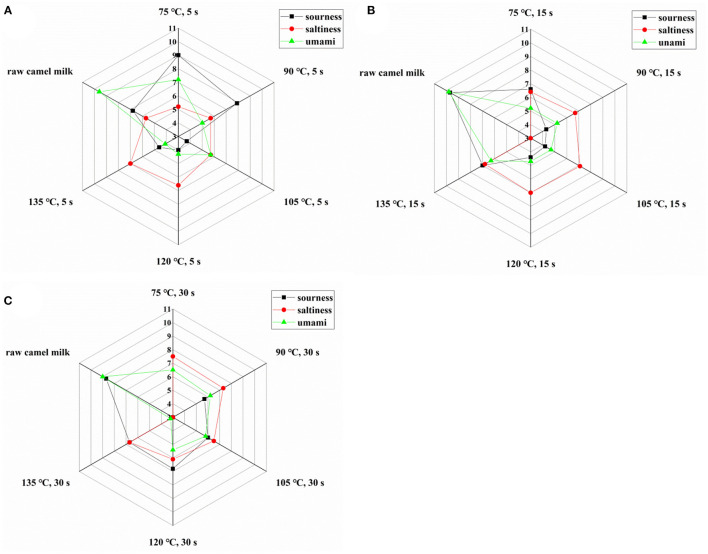
Taste index of camel milk when heated at different temperatures for 5 s **(A)**, 15 s **(B)**, and 30 s **(C)**.

The electronic tongue is equipped with five sensors that can evaluate the three tastes of sour, salty and umami respectively. As can be seen from Figure 3, compared with raw camel milk, the umami and sour taste of heated camel milk decreased and saltiness increased. The possible reason for the change of flavor was the decomposition of protein into free amino acids or the Maillard reaction between protein and lactose during the heating process, which greatly changed the taste of fresh milk. In addition, the hydrolysis of fat after heat treatment will affect the sour taste of fresh milk, and sodium salt will play a certain role in saltiness (31).

### 3.4. Volatile compounds detected using HS-GC-IMS

**Figure 4:**
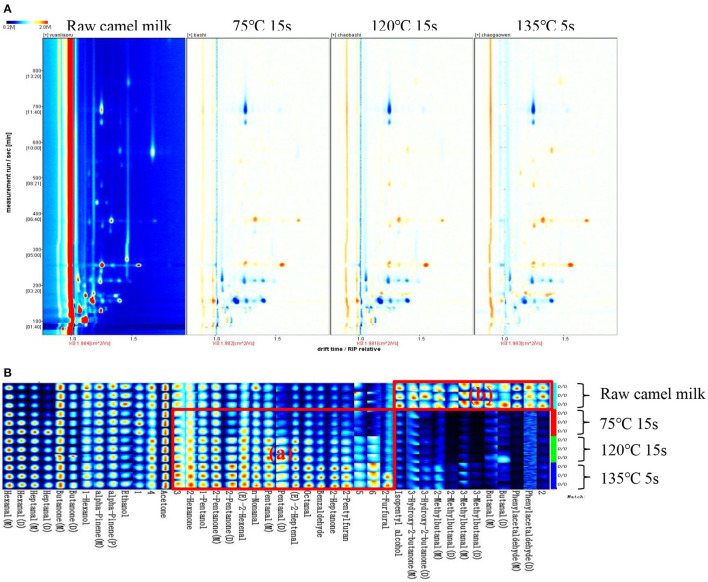
GC-IMS spectra **(A)** and gallery plot **(B)** of volatile organic compounds in camel milk of 4 different heat treatments.

HS-GC-IMS collected and analyzed only raw camel milk, heated at 75°C for 15 s, 120°C for 15 s and 135°C for 5 s. This is because these three heating temperatures are commonly used for commercially available dairy products and it was desired to mainly observe the differences in volatile compounds between these three temperatures and raw camel milk. The spectrogram of raw camel milk was selected as the reference, and the spectrogram of other samples was deducted as the reference. When the volatile organic compounds are the same, the background after deduction is white, while red means that the concentration of the volatile organic compounds is higher than the reference ratio, and blue means that the concentration of the volatile organic compounds is lower than the reference ratio. It is obvious from Figure 4A that there are significant differences in volatile organic compounds in camel milk with four different heat treatments. To compare specific volatile flavor compounds in each group of samples, all peaks were selected for fingerprint comparison (Figure 4B). The rows represent the samples to be tested, and the columns represent the content of the same volatile substance in different samples. The individual dots represent a volatile substance, and the color range represents the level of volatile substance, with the brighter the color, the higher the concentration. The integrated peak areas of the measured volatile substances are shown in Table 2. In fingerprints, unidentified substances are represented by numbers, and some monomer, dimer and polymeric forms were detected.

**Table 2:** Comparison of integrated peak area of volatile compounds in camel milk of different heat treatments.

**Compound**	**RI**	**Rt [sec]**	**Dt [a.u.]**	**Volume(a.u.)**			
				**Raw camel milk**	**75°C 15s**	**120°C 15s**	**135°C 5s**
n-Nonanal	1,102	767.555	1.48074	301.55 ± 63.85	284.63 ± 8.20	329.55 ± 10.65	367.10 ± 13.12
Phenylacetaldehyde (M)	1,066.7	691.291	1.25216	1,065.51 ± 31.45	153.30 ± 21.93	127.45 ± 5.40	120.37 ± 8.12
Phenylacetaldehyde (D)	1,066.7	691.291	1.54108	52.16 ± 6.97	26.52 ± 6.24	28.87 ± 2.94	25.39 ± 2.91
Octanal	1,005.8	577.239	1.40394	196.33 ± 8.89	215.28 ± 12.07	327.26 ± 15.18	388.91 ± 10.58
2-Pentylfuran	991.3	550.282	1.25664	37.50 ± 1.03	42.58 ± 8.41	58.49 ± 2.23	87.67 ± 6.03
Benzaldehyde	972.9	512.266	1.15201	150.09 ± 8.42	142.66 ± 6.91	179.97 ± 1.48	280.72 ± 1.64
(E)-2-Heptenal	955.9	479.681	1.25805	163.60 ± 13.13	185.69 ± 10.95	288.63 ± 3.57	292.59 ± 3.53
Heptanal (M)	898.2	383.406	1.33441	634.11 ± 33.22	1,022.33 ± 222.96	1,013.66 ± 12.09	1,155.57 ± 9.13
Heptanal (D)	897.5	382.419	1.69921	98.19 ± 4.01	253.95 ± 113.92	247.68 ± 11.21	319.29 ± 2.21
2-Heptanone	886.5	367.114	1.2623	179.66 ± 13.96	209.56 ± 18.89	281.07 ± 5.01	372.36 ± 2.20
(E)-2-Hexenal	845	317.248	1.18594	90.12 ± 2.45	104.76 ± 7.26	121.45 ± 5.76	126.02 ± 3.31
Hexanal (M)	785.8	257.428	1.25259	1,488.83 ± 59.25	1,810.35 ± 113.09	1,916.23 ± 4.72	1,930.60 ± 11.17
Hexanal (D)	784.5	256.298	1.56002	677.59 ± 53.66	1,224.46 ± 275.28	1,359.69 ± 43.90	1,414.09 ± 11.103
1-Pentanol	766.7	240.479	1.25584	239.90 ± 10.48	213.68 ± 15.08	267.33 ± 3.08	294.56 ± 5.21
2-Hexanone	775	247.743	1.18547	84.68 ± 1.23	90.98 ± 6.58	95.75 ± 1.00	101.72 ± 1.30
Isopentyl alcohol	730.6	211.32	1.24358	670.68 ± 36.03	254.11 ± 2.34	229.06 ± 6.17	227.23 ± 1.07
2-Pentanone (M)	665.4	171.254	1.12736	703.50 ± 13.74	655.13 ± 10.13	780.53 ± 7.87	833.85 ± 2.17
2-Pentanone (D)	668.8	172.772	1.37047	254.54 ± 12.10	197.70 ± 4.11	322.30 ± 9.30	429.82 ± 4.14
2-Methylbutanal (M)	643.3	161.845	1.17361	975.23 ± 22.29	229.24 ± 5.57	289.83 ± 17.09	519.60 ± 14.19
2-Methylbutanal (D)	646.3	163.059	1.39774	221.81 ± 31.36	10.96 ± 1.24	15.64 ± 3.03	48.44 ± 4.83
3-Methylbutanal (M)	620	152.435	1.1831	1,143.12 ± 61.81	130.20 ± 5.50	200.69 ± 24.63	425.81 ± 31.38
3-Methylbutanal (D)	630.7	156.685	1.40604	295.34 ± 42.57	9.98 ± 1.143	16.48 ± 1.95	47.35 ± 5.22
Butanone (M)	547.7	126.636	1.07519	2,379.78 ± 82.82	2,092.71 ± 28.26	2,179.01 ± 118.99	2,311.45 ± 23.65
Butanone (D)	547.7	126.636	1.25544	899.38 ± 80.85	700.83 ± 30.34	829.56 ± 148.45	915.49 ± 51.25
Butanal (M)	586	139.687	1.10721	143.16 ± 10.86	66.11 ± 6.30	72.23 ± 7.55	62.49 ± 13.42
Butanal (D)	583.4	138.777	1.2922	57.82 ± 12.17	15.83 ± 0.32	28.96 ± 16.82	20.49 ± 3.92
Acetone	469.3	103.567	1.11551	10,053.62 ± 145.50	9,709.94 ± 263.74	9,573.71 ± 206.12	9,961.62 ± 282.23
1-Hexanol	874.3	351.759	1.32213	120.19 ± 14.91	88.08 ± 10.40	85.22 ± 7.16	97.76 ± 3.50
alpha-Pinene (M)	928.4	431.048	1.21681	308.80 ± 21.45	294.88 ± 8.13	300.99 ± 4.81	287.12 ± 5.081
alpha-Pinene (P)	928.8	431.803	1.29513	86.76 ± 9.35	81.53 ± 6.39	86.75 ± 2.12	73.62 ± 2.98
Pentanal (M)	680.7	178.109	1.18926	373.34 ± 15.52	437.10 ± 46.97	593.26 ± 26.96	570.09 ± 9.91
Pentanal (D)	684.9	180.025	1.42352	22.82 ± 2.43	25.11 ± 5.26	59.64 ± 8.713	64.35 ± 2.57
3-Hydroxy-2-butanone (M)	737.3	216.427	1.05591	601.91 ± 57.32	334.35 ± 44.571	320.16 ± 17.47	362.42 ± 26.97
3-Hydroxy-2-butanone (D)	735.2	214.785	1.32982	57.21 ± 10.98	15.35 ± 1.86	18.22 ± 0.62	25.69 ± 3.29
Ethanol	435.2	94.904	1.0499	141.07 ± 5.66	111.19 ± 10.38	91.49 ± 5.95	102.80 ± 6.80
2-Furfural	822.9	293.456	1.08462	51.52 ± 3.46	45.95 ± 1.58	45.14 ± 1.26	67.18 ± 16.65

The fingerprint analysis clearly shows the differences in volatile organic compounds in different heat treatments of camel milk, and also labels the characteristic volatile organic compounds in each sample. As shown in Figure 4, furfural, 2-n-pentylfuran, 2-heptanone, benzaldehyde, octanal, trans-2-heptenal, valeraldehyde, nonanal, trans-2-hexenal, 2-pentanone, 1-pentanol and 2-hexanone were detected in the heat-treated camel milk samples, which were labeled as (a) region and were characteristic volatile organic compounds in the fingerprint.

Phenylacetaldehyde, butyraldehyde, 3-methyl-butyraldehyde, 2-methyl-butyraldehyde, 3-hydroxy-2-butanone and isopentyl alcohol were detected in raw camel milk samples, which were labeled as the (b) region of characteristic volatile organic compounds in the samples. As shown in Figure 4, aldehydes and ketones in camel milk changed greatly before and after heating. Aldehydes, ketones and organic acids are the main products of Maillard reaction, among which ketones are mainly the synthesis products of the β oxidation reaction of saturated fatty acids, which are important flavor substances in dairy products with milk flavor and sweet flavor (32–34). Aldehydes may be secondary or tertiary oxidation products of milk fat oxidation. Because of their low flavor threshold and obvious flavor characteristics, aldehydes are also important components of milk volatile substances (35, 36).

## 4. Conclusion

With the increase of heat treatment temperature and time, the contents of FRS and 5-HMF increased significantly, and when the heat treatment temperature was higher than 120°C, the contents of FRS and 5-HMF increased greatly, and reached the maximum value when the heating temperature reached 135°C. The FRS and 5-HMF contents were also compared with those of cow's milk, and the results showed that the FRS content of cow's milk was generally higher than that of camel milk at the same heat treatment conditions, while the 5-HMF content of camel milk was generally higher than that of cow's milk. The flavor and taste of camel milk will change with the increase of heat treatment degree. Compared with raw milk, the content of aldehydes and ketones increased with the increase of heat treatment. Through the above research, we believe that the heating condition of 120°C for 15s is better. This study can provide reference for exploring the Maillard reaction degree and volatile flavor substance changes of camel milk with different heat treatment degrees. However, there are many limitations in this study, for example, the samples were only selected from camel milk in Alashan region and the samples were also mature milk, and the results are not representative of the variation in all camel milk.

## Data availability statement

The original contributions presented in the study are included in the article/supplementary material, further inquiries can be directed to the corresponding authors.

## Author contributions

XZ: validation, investigation, data curation, formal analysis, and writing—original draft. YG: formal analysis. YZ and XP: writing—review and editing. YW: visualization. SZ and JL: writing—review and editing, project administration, and funding acquisition. All authors contributed to the article and approved the submitted version.
